# The Cognitive-Enhancing Effects of *Dendrobium nobile* Lindl Extract in Sleep Deprivation-Induced Amnesic Mice

**DOI:** 10.3389/fpsyt.2021.596017

**Published:** 2022-01-19

**Authors:** Ning Jiang, Yu-jiao Li, Meng-di Wang, Hong Huang, Shanguang Chen, Yinghui Li, Lina Qu, Fengzhong Wang, Xinmin Liu, Qiong Wang

**Affiliations:** ^1^Sino-Portugal TCM International Cooperation Center, The Affiliated Traditional Chinese Medicine Hospital of Southwest Medical University, Luzhou, China; ^2^Research Center for Pharmacology and Toxicology, Institute of Medicinal Plant Development (IMPLAD), Chinese Academy of Medical Sciences and Peking Union Medical College, Beijing, China; ^3^National Key Laboratory of Human Factors Engineering/State Key Laboratory of Space Medicine Fundamentals and Application, Chinese Astronaut Research and Training Center, Beijing, China; ^4^Institute of Food Science and Technology, Chinese Academy of Agricultural Sciences (CAAS), Beijing, China

**Keywords:** *Dendrobium nobile* Lindl, sleep deprivation, learning and memory, neurotransmitters (5-HT and NE), oxidative stress markers 3

## Abstract

Chronic sleep deprivation (SD) causes neurological and neurodegenerative dysfunction including learning and memory deficit. The orchid *Dendrobium nobile* Lindl (DNL), is widely used as a Yin tonic and medicinal food throughout Asia, and has many reported pharmacological effects. This study focused on the cognitive-enhancing effects of DNL in sleep deprivation-induced amnesia in mice and its biochemical mechanisms. Our results showed that the mice displayed significant cognitive deficits after 2-week SD while treatment with the extract of DNL prevented these impairments. In the novel object recognition and object location recognition tasks, a significant increase in the discrimination index was observed in DNL-treated (200 and 400 mg/kg) mice. In the MWM test, DNL (200 and 400 mg/kg) treatment shorten the prolongation of latency and increased the crossing numbers compared with SD mice. The biochemical analysis of brain tissue showed a decrease in NE, dismutase (T-SOD) and catalase (CAT) activity and an increase in 5-HT and malondialdehyde (MDA) concentration after the treatment with DNL in mice. Our findings indicated that DNL exerted a positive effect in preventing and improving cognitive impairment induced by SD, which may be mediated via the regulation of neurotransmitters and alleviation of oxidative stress.

## Introduction

Chronic sleep deprivation is a common problem, and it has been estimated that 50–70 million adults in the United States have sleep or wakefulness disorder (1). Sleep loss contributes to neurological and neurodegenerative disorders including memory deficits which are increasingly regarded as a major public health and safety issue (2) and financial and social burden. Cognitive enhancers including as modafinil and donepezil, and stimulants such as caffeine and nicotine have been reported to prevent the memory impairment induced by chronic sleep deprivation (SD), but many have undesirable side-effects and cause habituation (3). Safer and more effective treatments for memory impairment induced by SD are thus under investigation.

**Figure 1:**
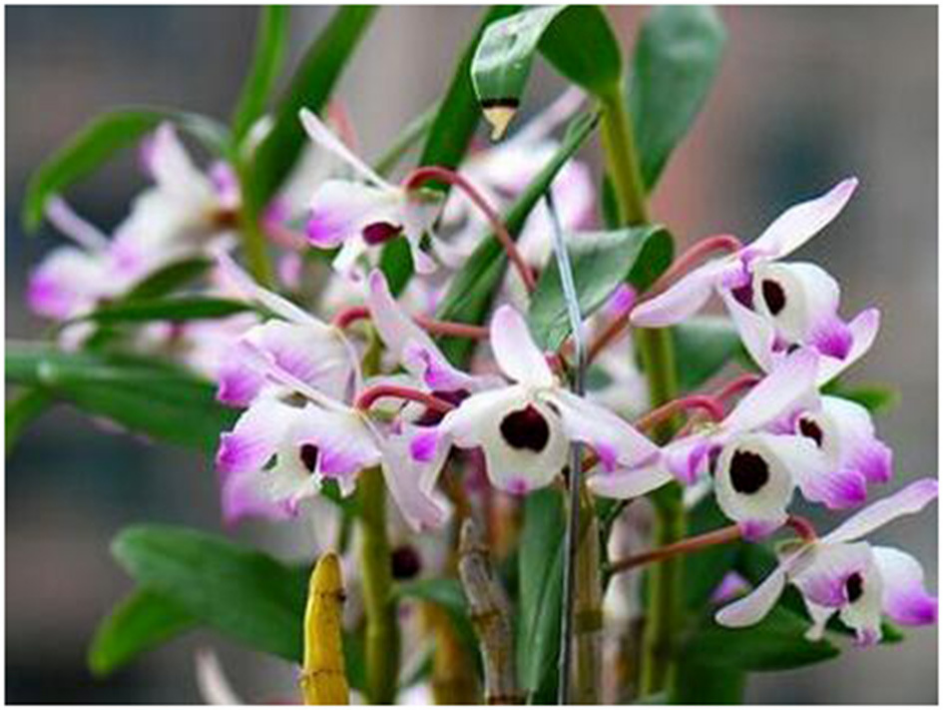
The picture of DNL.

*Dendrobium nobile* Lindl (DNL) is a precious traditional Chinese medicine, it's an epiphytic orchid distributed throughout tropical and subtropical Asia (N. E India, China, Malaysia, Japan) which is used a tonic and medicinal food (Figure 1). The main chemical components of DNL are alkaloids, polysaccharides, amino acids, phenols, volatile oils, etc. The DNL has a wide range of pharmacological effects (4, 5). Recently researches focused on its neuroprotective and cognitive-enhancing effects (6, 7), and its effect on improving nerve cell damage (8). It was reported that the alkaloids and polysaccharide in DNL have obvious protective effects on LPS-induced learning and memory impairment in rats (9). DNL also has displayed ameliorative effects on memory impairment induced by lipopolysaccharide and Aβ_25−35_ in rats, but its activity against memory impairment induced by sleep deprivation has not been reported (10, 11). The present study was designed to investigate the effect of DNL extract on learning and memory in a sleep deprivation model and its underlying mechanisms, to explore its potential application in treating SD-induced impairment.

## Materials and Methods

### Drugs

Donepezil hydrochloride were purchased from Eisai (Ibaraki, Japan). Norepinephrine (NE) from the National institutes for Food and Drug Control (Beijing, China); dopamine (DA), 5-hydroxytryptamine (5-HT), 5-HIAA (5-hydroxyindole acetic acid), GABA (Gamma amino acid butyric acid) and DOPAC (Dihydroxy- phenyl acetic acid) from Sigma-Aldrich Co. (St. Louis, MO, USA) and Superoxide Dismutase (SOD), Catalase (CAT) and Malondialdehyde (MDA) commercial kit were from Nanjing Jiancheng Biotechnology Institute (Nanjing, China). Air-dried stems of DNL were purchased from Luzhou in SiChuan province and further authenticated by professor Bengang Zhang (Institute of Medicinal Plant Development, Beijing, China) according to their macroscopic characteristics. The air-dried stems of DNL were crushed and soaked in 80% acidic ethanol for 1 night. The extract was re-extracted three times with boiling acidic ethanol. After filtration, the solution was concentrated by vacuum-rotary evaporation at 60°C and then freeze-drying 12 h, The extraction value was 14%.

### Analysis

DNL was characterized chromatographically under the following conditions: Waters Acquity UPLC HSS T3 (1.8 um, 2.1 × 100 mm); flow rate: 0.3 ml/min, column temperature: 30°C; mobile phase A, 0.1% formic acid aqueous solution; phase B acetonitrile as a gradient elution. The chemical profile is shown in Table 1.

**Table 1:** Chemical profile of *Dendrobium nobile* extract (DNL).

**Number**	**Peak area**	**Compound**	**Molecular formula**
1	2805.415137	-	-
2	100197.7715	Ficusal-4-O-β- d -glucopyranoside	C24H28O11
3	144967.1248	-	-
4	22285.69431	dendroside G	C21H34O10
5	11267.58756	dendrobin A	C16H18O4
6	14866.99716	dendronobiloside A	C27H48O12
7	686.8800255	dendronobiloside C	C27H44O12
8	33576.83579	-	-
9	258.3121648	-	-
10	75152.12667	Citrusin C	C16H22O7
11	87059.25145	Trans-methyl cinnamate-2-O-beta-D-glucoside	C16H20O8
12	108334.4155	-	-
13	23859.5398	Zhepiresinol	C14H16O6
14	21912.22788	-	-

### Animals and Treatments

72 ICR male mice (20–22 g) (Institute of the Chinese Academy of Medical Science Center, Beijing, China) were housed under standard conditions for 3 days prior to testing to adapt to the new environment. All experiments were conducted according to the “Principles of Laboratory Animal Care” (NIH publication No.86-23 1996) and P.R. China legislation. The protocols were approved by the committee for the Care and Use of Laboratory Animals of IMPLAD, CAMS & PUMC, China (NO. 20161028), all experiments adhered to standard biosecurity and institutional safety procedures.

Animals were divided into 6 groups: the control group, the SD model group, the DNL-treated groups (100, 200, and 400 mg/kg) and the donepezil group (3 mg/kg). The doses of DNL and Donezepil were based on our previous study (12). DNL and donepezil were administrated orally once a day to mice for 14 days before SD until the end of experiment. The control group and the SD model group were given the same volume of distilled water, and except for the control group, other mice were exposed to SD for 14 days.

**Figure 2:**
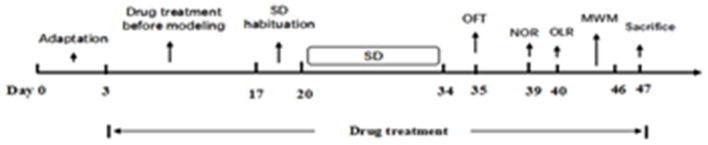
The experimental procedure.

After habituation for 3 days, all except the control group were subjected to SD from 8 a.m. to 11 a.m. each day. Then all animals were moved back to the housing room. The process lasted for 2 days in order that the mice could acclimatize. Following the induction period, all test groups were exposed to SD for 14 days. After a 2-week SD exposure, behavioral changes tests were conducted as follows: open field test (OFT), novel object recognition task (NOR), object location recognition task (OLR), and Morris water maze test (MWM). Animals were sacrificed after the behavior tests and blood and brain tissue were taken for analysis (Figure 2).

### The SD Mice Model

The SD procedure was performed as our previously studies in our lab (3, 13, 14). Briefly, except the mice in the control group, other mice were placed in Sleep Deprivation Apparatus (developed by the Institute of Medicinal Plant Development, Chinese Academy of Medical Sciences, and the Chinese Astronaut Center, patent No. 201210356645.X) for 2 weeks continuously. Before encountered to the SD procedure, the mice receipt 3 days' adaptation (3 h per day, during 12:00 p.m.−15:00 p.m.). The speed of SD apparatus was 60 s per rotation and there was a 2 min pause between two rotations. After finishing the adaptation, the mice were put into the SD apparatus and suffered for 14 consecutive days' SD modeling, then the behavioral tests were tested in mice. The mice were free to have water and food during the SD experiments.

### Behavioral Tests

#### Open-Field Test

The open field test was conducted on day 15. The apparatus consists of four metal tanks (diameter 30 cm, height 40 cm) with a video camera fixed at the top (15). For each experiment, four mice were place in the center of each tank and their locomotor activities in 10 min were detected. The total distance was recorded as the index of the locomotive activity in mice.

#### Novel Object Recognition Task

This task consists of three phases: habituation, familiarization, and test. In the habituation phase, animals were placed individually in a square metal box (black, 60 × 40 × 80cm) with no objects in it, for 10 min in three consecutive days. On day 4, in the familiarization phase, two identical objects (A1 and A2) were placed on opposite sides of the box, and the animal was placed in the box for 5 min to explore the two objects. After 20 min in the home cage, the test was performed and the mice placed in the same metal box and presented with two objects, the old familiar A1, and a new object B, to replace object A2 for 5 min. The discrimination index (DI) was calculated as the percentage of time spent exploring the novel object over the total time spent exploring the two objects (16).

#### Object Location Recognition Task

The Object Location Recognition (OLR) task is an accepted method for testing spatial location memory. The protocol was similiar as the novel object recognition test. Instead of replacing one of the original identical objects (A1 and A2), A1 or A2 was moved to a new location (17). The discrimination index (DI) was calculated as before (18).

#### Morris Water Maze Test

The Morris Water Maze test was performed on the 21st day of SD to further investigate the effects of DNL on spatial memory in mice. The water maze is a circular pool (100 cm in diameter, 40 cm in high), with the water (23–25°C) made opaque with black ink. An “invisible” platform (metal, black, 6 cm in diameter, 15 cm in high) was placed 1.5 cm below the water surface, providing the only outlet channel. The protocol was described in our previous studies with minor modifications (19–21).

##### Escape Acquisition

The mice were subjected to three trials per day for 5 days. At the beginning of the test, the mice were trained to remember the platform by being placed on it for 10 s, before being released into the water, facing the wall, at a different starting point each time, and allowed to swim for a maximum of 90 s in each trial. If they failed to escape within 90 s, the mice were gently guided to the platform and allowed to stay there again to learn, and the escape latency was recorded as 90 s.

##### Probe Trial

After the escape acquisition test, each animal was subjected to the probe trial, in which the platform was absent. The mice were released from the opposite quadrant where the platform had been located and allowed to swim and explore the pool for 90 s. Swimming distance in the target quadrant and the number of target crossings were recorded as measures for spatial memory.

### Biochemical Analysis

The levels of neurotransmitters were determined in the brain tissues in rats described as below:

#### Brain Tissue Extraction

After the behavioral tests, mice were sacrificed by decapitation. The brains were removed and placed on ice, and the hippocampus and cerebral cortex tissues were dissected out rapidly, weighed, and stored at −80°C.

#### Measurement of Neurotransmitter Levels in the Hippocampus

Hippocampus tissues were homogenized and mixed with acetonitrile containing the internal standard (5 μg/mL, 3, 4-dihydroxybenzylamine, DHBA). After centrifuging at 4°C (20,000 rpm, 30 min), the supernatant was collected, and neurotransmitter levels measured by liquid chromatography-tandem mass spectrometry. Chromatographic separation was performed on a TSK Gel amide 80 column (2.0 mm × 15 cm, 3 μm); column temperature 35°C; mobile phase system acetonitrile-ammonium formate solution (15 mmol·mL-1, pH = 5.5) (40: 60), flow rate 0.4 mL·min^−1^; operated under the multiple reaction monitoring (MRM) mode using electrospray ionization (ESI) in the positive ion mode, at m/z 177.0 → 160.0 (5-HT), 170.0152.0 (NE) and 140.0 → 123.0 (DHBA as internal standard). Neurotransmitter concentrations were quantified using peak area ratios vs. internal standard.

#### Detection of Brain T-SOD, CAT Activities, and MDA Levels

The hippocampus and cerebral cortex were each homogenized in 10 volumes of cold saline. After centrifuging at 4°C (2,500 rpm, 10 min), the supernatants were collected. The total protein content of sample was estimated with the Pierce BCA Assay kit, using bovine serum albumin as the standard (22). T-AOC and CAT activity and MDA levels were determined according to the manufacturer's protocols (Jiancheng Institute of Biotechnology, Nanjing, China).

### Data and Statistical Analyses

Statistical analysis was carried out using SPSS 17.0 software (Chicago, IL, USA). All data are expressed as mean ± SEM. Data recorded from the acquisition trials of the MWM among the groups over a period of 5 days were analyzed by using repeated-measure two-way ANOVA. Other data were analyzed by one-way ANOVA followed by multiple *post hoc* comparisons using the LSD test. Statistical significance was defined as *p* < 0.05 or *p* < 0.01.

## Results

### Effects of DNL in the Open-Field Test

**Figure 3:**
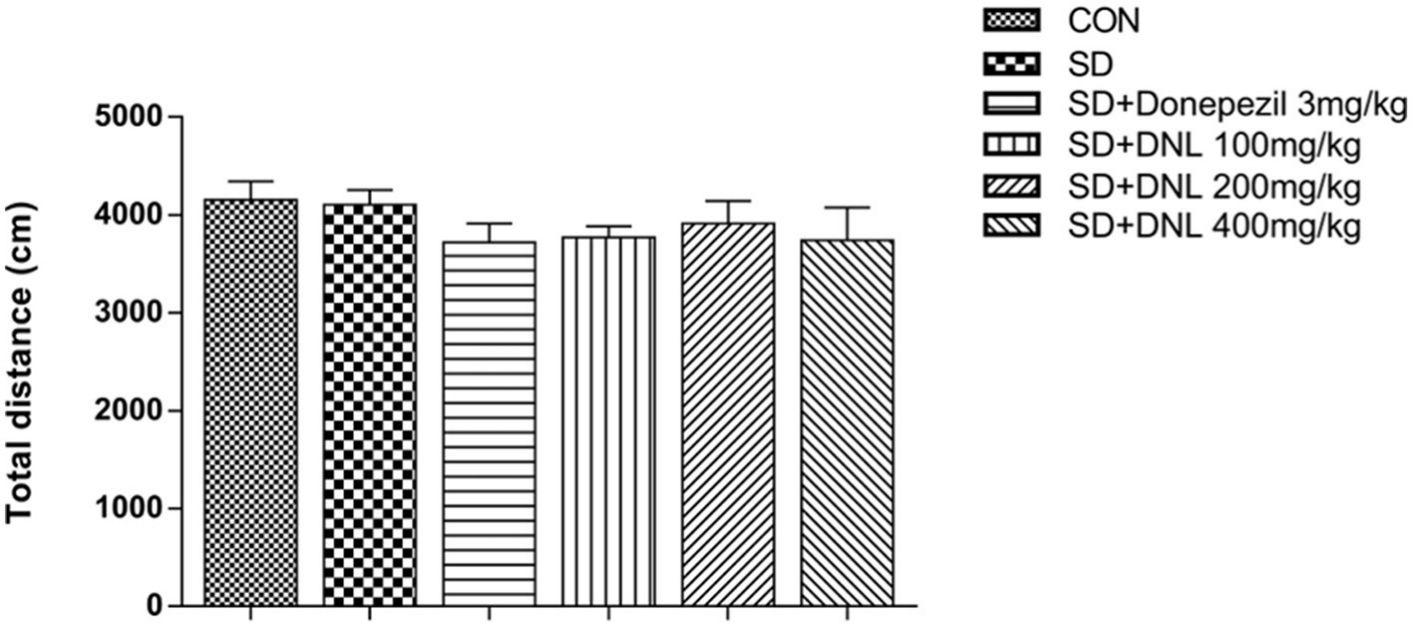
The effect of DNL on the locomotor activities in the open field test after SD for 2 weeks in mice. Values are mean ± SEM, *n* = 10–12.

The total distance is used to indicate the locomotive activity after 2-week SD. Figure 3 showed there was no significant difference between the six groups.

### Effects of DNL in the NOR Task

**Figure 4:**
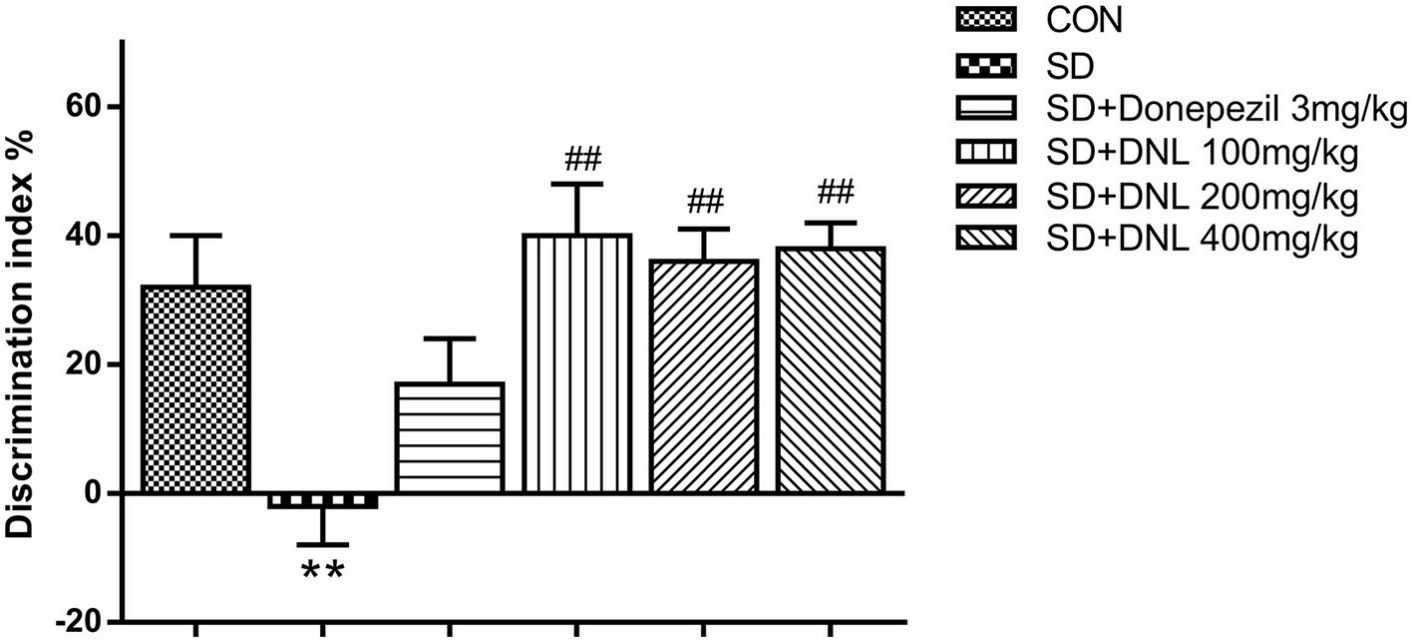
The effect of DNL on the discrimination index (DI) during the testing session in the NOR task after SD for 2 weeks in mice. Values are mean ± SEM, *n* = 10–12. **p < 0.01 vs. the Con group; ^##^*p* < 0.01 vs. the SD group.

After 2-week SD, in the familiar phase, the mice spent comparable time on exploring the two similar objects, with no preference between positions of the objects. During the test phase, the DI significantly decreased in the SD model group in comparison with the control group. Compared with the SD group, the DI of the three DNL-treated groups showed a significant decrease (*p* < 0.05, Figure 4).

### Effects of DNL in the OLR Task

**Figure 5:**
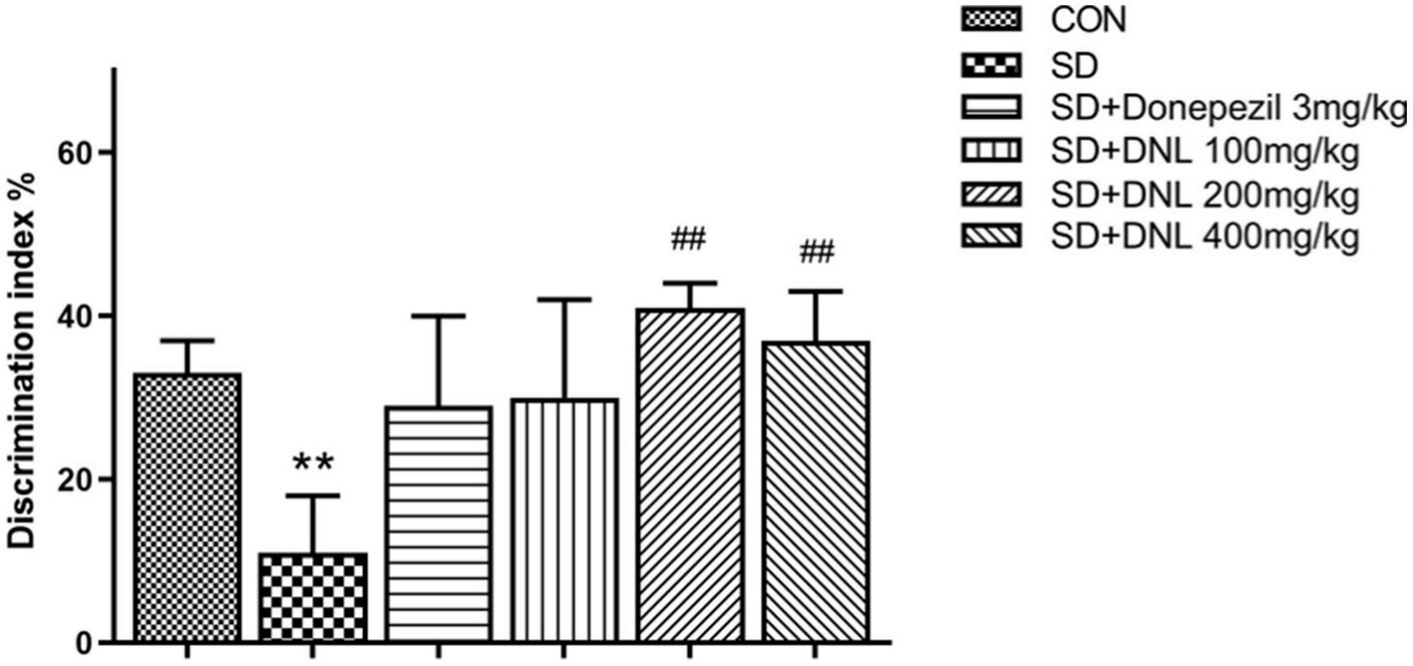
The effect of DNL on the discrimination index (DI) during the testing session in the OLR task after SD for 2 weeks in mice. Values are mean ± SEM, *n* = 10–12. **p < 0.01 vs. the Con group; ^##^*p* < 0.01 vs. the SD group.

Be similiar with the NOR, mice in the OLR task showed no difference in preference in the familiarization phase. In the test phase, the control mice spent significantly longer exploring on the object in the new location compared with the SD mice. DNL treatment (200 and 400 mg/kg) significantly increased DI compared to the SD group (*p* < 0.01; Figure 5), however the donepezil group (3 mg/kg) showed no significant difference compared to the SD group (*p* > 0.05).

### Effects of DNL in the MWM Test

**Figure 6:**
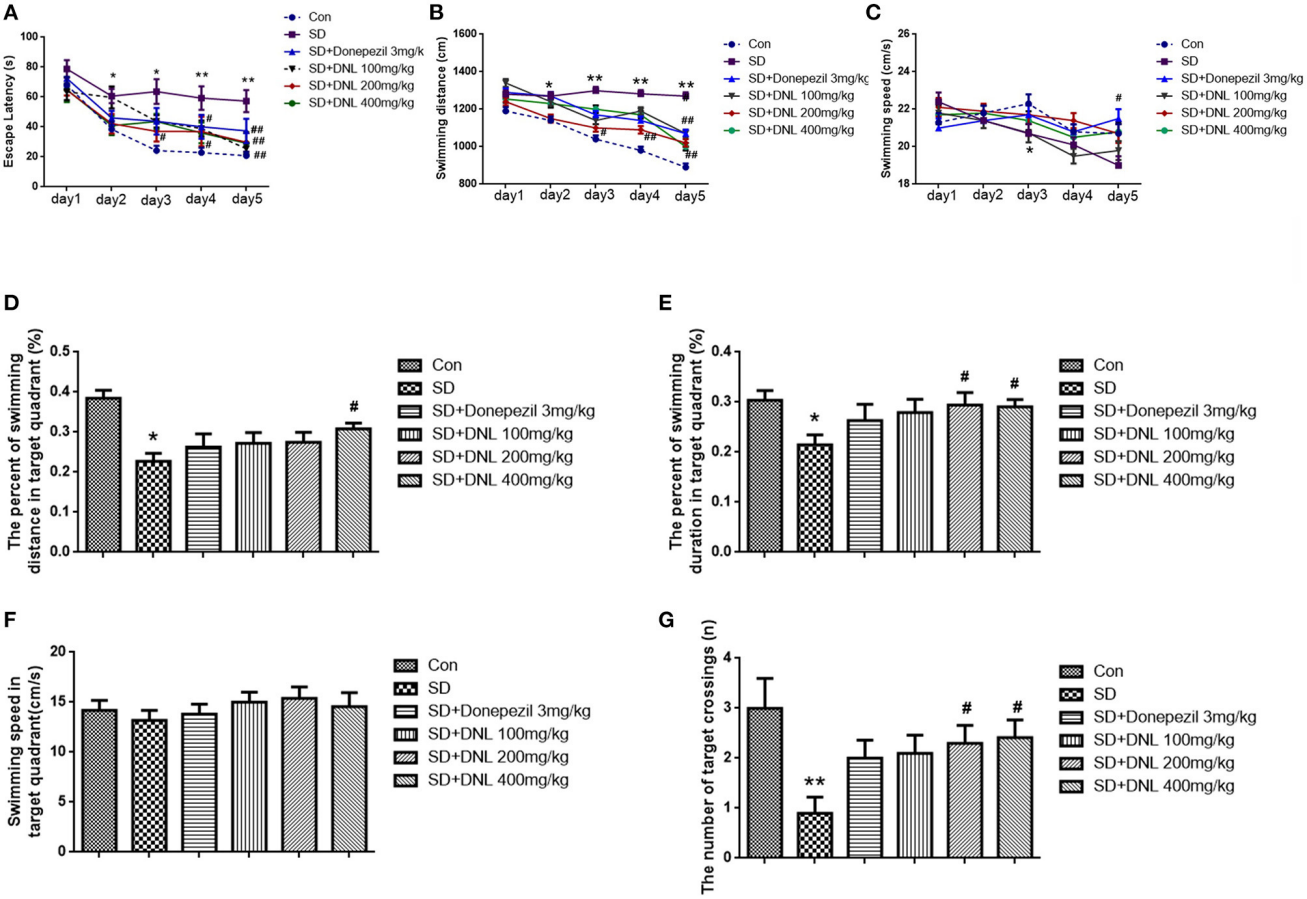
The effect of DNL on the Escape Latency **(A)**; Swimming distance **(B)**; Swimming speed **(C)**; The percent of swimming distance in target quadrant **(D)**; The percent of swimming duration in target quadrant **(E)**; Swimming speed in target quadrant **(F)**; The number of target crossings **(G)** in the MWM test after SD 2 weeks in mice. Values are mean ± SEM, *n* = 10–12. *p < 0.05 vs. the Con group, **p < 0.01 vs. the Con group; ^#^*p* < 0.05 vs. the SD group, ^##^*p* < 0.01 vs. the SD group.

The Morris water maze task was conducted over 6 days, ending with the probe test on the last day. As shown in Figures 6A–C, in the escape acquisition phase, the escape latency and the swimming distance in SD mice was higher than the control group from the second day (*p* < 0.05; *p* < 0.01). The swimming speed in the SD group was decreased from day 3 (*p* < 0.05). DNL at a dose of 200 mg/kg attenuated the spatial learning deficits, as observed by a reduction of the escape latency and swimming distance from day 3 to day 5 (*p* < 0.01; *p* < 0.05). Similarly, the high-dose group (400 mg/kg) on days 4 and 5 reduced escape latency compared to the SD mice. Donepezil (3 mg/kg) reduced the escape latency and the swimming distance and increased the swimming speed on the 5th day (*p* < 0.01; *p* < 0.05).

In the probing phase (Figures 6D–G), SD rats showed a decreased percentage in swimming distance and swimming duration in the target quadrant (*p* < 0.05) compared to the control rats, whereas DNL (200, 400 mg/kg) mitigated the trend. The swimming speed in the target quadrant was no different in all groups. The number of SD rats crossing through the platform where had previously been located was significantly reduced compared with the control rats (*p* < 0.01). With the treatment of DNL (200, 400 mg/kg), the crossing number was increased compared with the SD group (*p* < 0.05).

### Effects of DNL on 5-HT and NE in the Hippocampus

**Figure 7:**
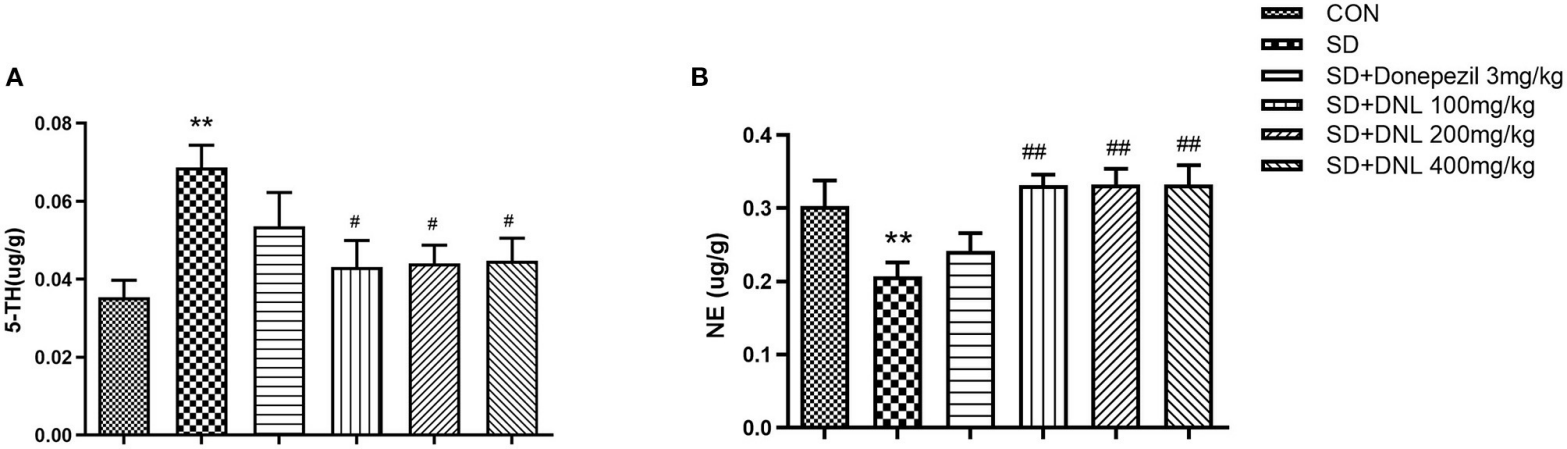
The effect of DNL on 5-hydroxytyptamine (5-HT) **(A)** and norepinephrine (NE) **(B)** levels in the hippocampus after SD 2 weeks in mice. Values are mean ± SEM, *n* = 8. **p < 0.01 vs. the Con group; ^#^*p* < 0.05 vs. the SD group, ^##^*p* < 0.01 vs. the SD group.

The neurotransmitter content of the hippocampus after 44 days of DNL treatment is shown in Figure 7. After SD induction, hippocampus level of 5-HT in SD mice were increased, while the concentration of NE was decreased compared to the control group (*p* < 0.01). Treatment with DNL (100, 200, and 400 mg/kg) decreased 5-HT level while elevating NE level in the hippocampus in SD rats (*p* < 0.05, *p* < 0.01, *p* < 0.05). No marked difference in 5-HT and NE levels detected in donepezil-treated SD mice.

### Effects of DNL on Oxidative Stress Markers in Hippocampus and Cerebral Cortex

**Figure 8:**
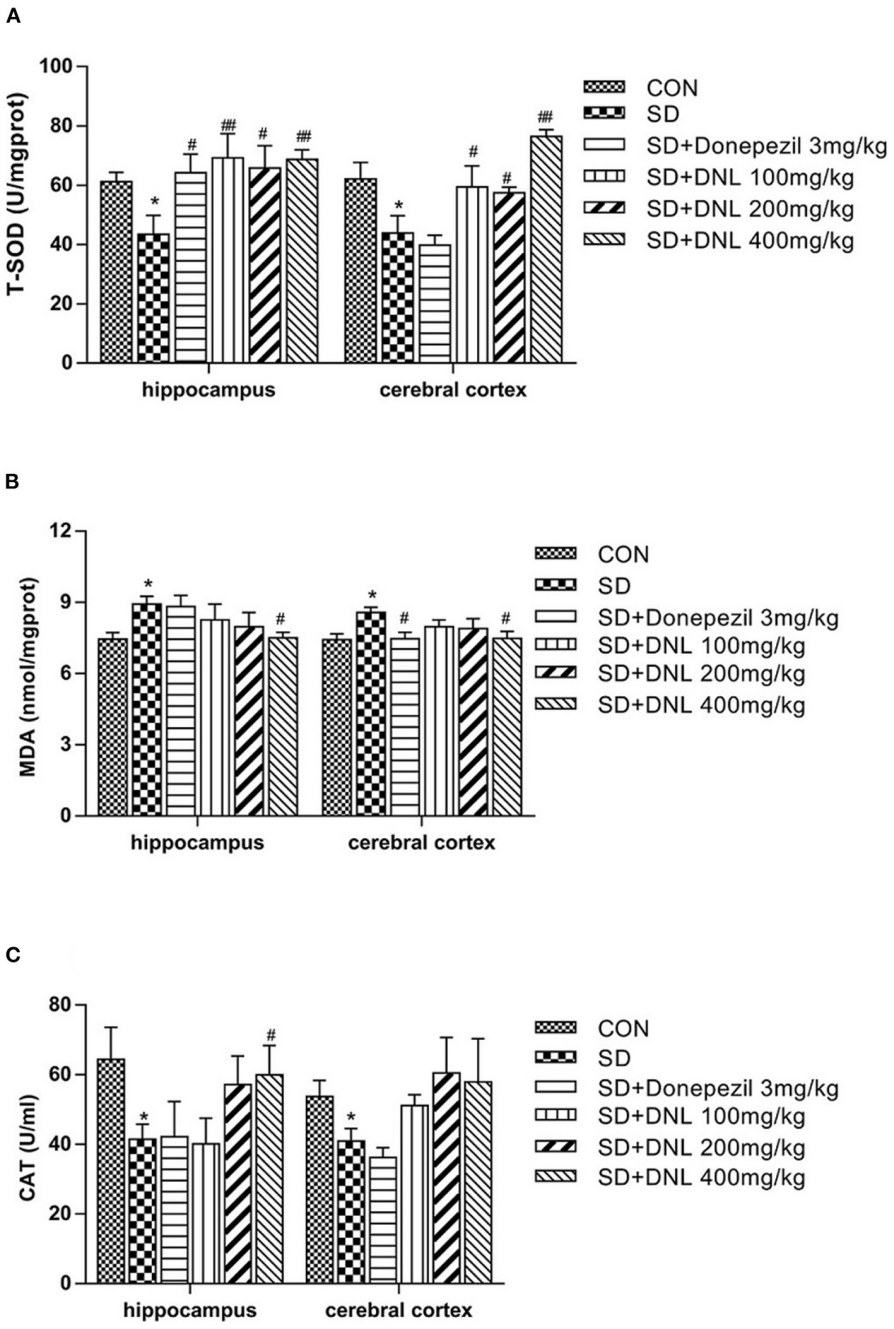
The effect of DNL on the activities of Total Superoxide Dismutase (T-SOD) **(A)**; Catalase (CAT) **(B)** and Malonaldehyde (MDA) **(C)** in the hippocampus and cerebral cortex of mice after SD for 2 weeks. Values are mean ± SEM, *n* = 8. *p < 0.05 vs. the Con group; ^#^*p* < 0.05 vs. the SD group, ^##^*p* < 0.01 vs. the SD group.

The T-SOD and CAT activities were significantly decreased, while MDA levels was markedly increased in both hippocampus and cerebral cortex in the SD group compared with the control group (*p* < 0.05) (Figure 8). Compared with the SD mice, brain T-SOD activities in all the DNL-treated groups were increased (*p* < 0.05, *p* < 0.01). DNL (400 mg/kg) treated mice showed a significant decrease in the level of MDA in both brain regions compared to the SD group (*p* < 0.05). Hippocampus CAT activities of DNL (400 mg/kg) group were increased compared to the SD mice (*p* < 0.05). Donepezil (3 mg/kg) treatment significantly increased hippocampus T-SOD activities and reduced cerebral cortex MDA level in the SD mice (*p* < 0.05).

## Discussion

### Sleep Deprivation Could Induce Cognitive Impairments

Sleeping plays a key role on human's health, especially for the brain health. Adequate sleep is basically imperative for memory consolidation (23). Accumulating evidence suggests that sleep deprivation produces could induce memory deficits, and in animals these can be assessed by using behavioral tests, including avoidance tasks (24), object recognition tasks and the Morris water maze task (25). Sleep deprivation as cognitive challenge may therefore provide a promising preclinical model of memory impairment and a useful tool to study cognition enhancing drugs (26). Previous studies exhibited that sleep deprivation could induce serious cognitive dysfunction (27, 28). In our experiment, we found SD 2 weeks in mice could induced obviously cognitive associated behavioral impairments, such as spatial and non-spatial learning and memory deficits. These results are consistent with previous studies.

### DNL Has No Sensorimotor Effect in the Open-Field Test in the SD Mice

Open-field test is mainly used to test the locomotor activities of animals in a relatively closed environment during a certain period of time, and widely used in animal experiment (29). The results of this study showed that after 2-week of SD, there was no significant difference between any two groups, which showed DNL had no influence on the locomotor activity in mice. This result indicated that the effect of DNL was originally mnemonic, rather than the sensorimotor effect.

### DNL Could Ameliorate Short-Term Memory in the ORT in the SD Mice

The object recognition task (ORT) is based on the principle of preference for novel objects in rodents and can assess recognition memory in the spontaneous state (30). The ORT takes advantage of the nature of preference for “novel” objects in rodents in the spontaneous state (31). During the experiment, the animals are in accordance with the characteristics, the location, the order of appearance, and the background of objects to distinguish the different “identity” objects, achieving the transition through detection of simple non-spatial memory to complex spatial, temporal and episodic memory (32). Based on the ORT, the object location recognition (OLR) and the novel object recognition (NOR) were established. The former was used to assess short-term, spatial memory, while the latter was designed to evaluate short-term, non-spatial memory. In agreement with the previous studies (33, 34), our results showed that SD damaged the normal performance of mice in ORT tasks. However, compared with the SD model group, the treatment of DNL effectively enhanced DI in both OLR and NOR tasks. For the first time, we have demonstrated that DNL administration could significantly ameliorate impaired short-term memory in SD mice.

### DNL Could Ameliorate Long-Term and Spatial Memory in the Morris Water Maze in the SD Mice

Morris water maze test was used to assess effects of DNL on the long-term, spatial learning and reference memory. In this task, two main parameters are essential for assessing the spatial learning and memory ability in mice. The first is the escape latency in the acquisition trial, which means that the mice must learn the accurate position of the platform and develop suitable swimming approaches to reach the same from the randomly chosen starting point within 90 s. The second is the virtual-platform crossing numbers when the platform was absent in probe trial, which is a key indicator assessed reference memory (35, 36). In our study, compared with the SD group, the treatment of DNL effectively shorten escape latencies after 2 days' training to find the hidden platform in the escape acquisition phase, which showed DNL has a strong effect on ameliorating the reference memory impairment in the SD mice. In the probe trial, we found DNL could effectively increase the number of target crossings in the SD mice. These results indicating that DNL treatment remarkably improved the long-term, spatial memory in the SD mice.

### DNL Could Modulate Memory Deficit via 5-HT and NE in the Hippocampus of SD Mice

Monoamine neurotransmitters play an important role in the learning and memory. 5-HT mediates processes involved in central nervous system fatigue, helping to control sleep (37, 38). NE plays a major role in the maintenance of REM sleep (39), and levels of NE in the brain have been shown to be closely related to memory. In the present study, chronical sleep deprivation induced an obvious elevation of hippocampus 5-HT levels and a marked decline of hippocampus NE levels in mice, which was consistent with the previous study (40, 41). DNL treatment at all doses significantly reversed the elevation of 5-HT and the decrease of NE in SD mice, indicating that the cognitive-enhancing effects of DNL might be at least in part due to modulation of these two major monoamine neurotransmitters.

### DNL Could Repair Oxidative Damage in the Hippocampus and Cortex of SD Mice

Oxidative stress plays a prominent role in the pathogenesis of several cognitive impairment processes. Oxidative stress occurs in brain tissues whenever there is increased generation of reactive oxygen species (ROS) and impaired antioxidant defense systems. Brain tissue in particular is more susceptible to the deleterious effects of ROS because of its high rate of oxygen consumption and reduced antioxidant defense systems (19). Excessive ROS production may result in oxidative damage to proteins, lipids, and DNA and eventually in apoptosis or cell death, but ROS levels are balanced by the action of antioxidant enzymes, including CAT, SOD, and glutathione oxide. Sleeping deprivation could weaken the free radical scavenging enzyme system and cause system imbalance, which further aggravates brain damage which ultimately results in learning and memory disorders (42, 43). The changed hippocampal synaptic protein GAP-43, SYP, PSD-95 levels, serum corticosterone levels, and neuroinflammation were found in sleeping deprivation related memory impairment mice (44). Studies have confirmed the abnormal alterations in MDA, SOD, and CAT activities associated with memory impairment after sleep deprivation (45, 46). Similar changes were exhibited in our study: a decrease of SOD and CAT activities and an increase of MDA level in hippocampus and cerebral cortex of SD mice. Treatment with DNL enhanced antioxidant defense in the brain by increasing SOD and CAT levels and reducing MDA concentration induced by SD, contributing to the cognitive enhancement effect of DNL.

## Conclusion

The present study shows for the first time the cognitive-enhancing effect of DNL in a chronic stress model induced by SD in mice. The improvement effect of DNL in SD mice may be partially due to regulate the neurotransmitters and mitigate oxidative stress levels in the brain. Our results also indicate that, as a traditional medicinal herbal plant, DNL may be used as a potential candidate agent in preventing and treating cognition impairment induced by stress.

## Data Availability Statement

The raw data supporting the conclusions of this article will be made available by the authors, without undue reservation.

## Ethics Statement

The animal study was reviewed and approved by Care and Use of Laboratory Animals of IMPLAD, CAMS & PUMC, China (No. 20161028).

## Author Contributions

NJ: for doing animal experiments and process all experimental data. Y-jL: assist in animal experiments and process the data. M-dW: provide some experimental equipment help. All authors contributed to the article and approved the submitted version.

## Funding

This work was supported by the Science & Technology Department of Sichuan Province (2019YFH0023), the opening foundation of the State Key Laboratory of Space Medicine Fundamentals and Application, Chinese Astronaut Research and Training Center (SMFA18K06), the National Key Research and Development Program of China (2016YFE0131800), Office of Sciences & Technology and Talent Work of Luzhou (2018LZXNY-ZK32), and the High - end Talents Recruitment Program (Liu Xinmin group) of Luzhou Municipal People's Government.

## Conflict of Interest

The authors declare that the research was conducted in the absence of any commercial or financial relationships that could be construed as a potential conflict of interest.

## Publisher's Note

All claims expressed in this article are solely those of the authors and do not necessarily represent those of their affiliated organizations, or those of the publisher, the editors and the reviewers. Any product that may be evaluated in this article, or claim that may be made by its manufacturer, is not guaranteed or endorsed by the publisher.

## Abbreviations

SD: sleep deprivation
DI: discrimination index
DNL: Dendrobium nobile Lindl
LC-MS/MS: liquid chromatography-tandem mass spectrometry
MWM: Morris water maze
NOR: novel object recognition
OLR: object location recognition.

## References

[1] Strand LB Carnethon M Biggs ML Djoussé L Kaplan RC Siscovick DS. Sleep disturbances and glucose metabolism in older adults: the cardiovascular health study. Diabetes Care. (2015) 38:2050–8. 10.2337/dc15-0137 26384390 PMC4613916

[2] William DSK. Effects of sleep deprivation on cognition. Prog Brain Res. (2010) 185:105–29. 10.1016/B978-0-444-53702-7.00007-5 21075236

[3] Lu C Shi Z Dong LM Lv JW Xu P Li YH. Exploring the effect of ginsenoside Rh1 in a sleep deprivation-induced mouse memory impairment model. Phytother Res. (2017) 31:763–70. 10.1002/ptr.5797 28244162

[4] Huang J Huang NQ Zhang MH Nie J Xu YY Wu Q. Dendrobium alkaloids decrease Aβ by regulating α- and β-secretases in hippocampal neurons of SD rats. PeerJ. (2019) 6:e7627. 10.7717/peerj.7627 31534855 PMC6733236

[5] Ma C Meng CW Zhou QM Peng C Liu F Zhang JW. New sesquiterpenoids from the stems of *Dendrobium nobile* and their neuroprotective activities. Fitoterapia. (2019) 138:104351. 10.1016/j.fitote.2019.104351 31476401

[6] Zheng XK Cao XW Feng WS Kuang HX. Current status in studies of *Dendrobium nobile* Lindl. Chinese N Drugs J. (2005) 7:4.

[7] Chen JW Ma H Huang XN Gong QH Wu Q Shi JS. Improvement of *Dendrobium nobile* Lindl. alkaloids on cognitive deficit in rats induced by lipopolysaccharides. Chin J Pharmacol Toxicol. (2008) 22:6. 10.3867/j.issn.1000-3002.2008.06.00223335300

[8] Li SY Zhou JX Xu SF Li J Liu J Lu YF. Induction of Nrf2 pathway by *Dendrobium nobile* Lindl. alkaloids protects against carbon tetrachloride induced acute liver injury. Biomed Pharmacother. (2019) 117:109073. 10.1016/j.biopha.2019.109073 31212129

[9] Bang YH. ChemInform abstract: phenanthrenes from *Dendrobium nobile* and their inhibition of the LPS-Induced production of nitric oxide in macrophage RAW 264.7 cells. ChemInform. (2010) 41. 10.1002/chin.201045217 20483604

[10] Nie J Tian Y Zhang Y Lu YL Li LS Shi JS. Dendrobium alkaloids prevent Aβ_25−35_-induced neuronal and synaptic loss via promoting neurotrophic factors expression in mice. PeerJ. (2016) 4:e2739. 10.7717/peerj.2739 27994964 PMC5157189

[11] Yang S Gong QH Wu Q Li F Lu YF Shi JS. Alkaloids enriched extract from *Dendrobium nobile* Lindl. attenuates tau protein hyperphosphorylation and apoptosis induced by lipopolysaccharide in rat brain. Phytomedicine. (2014) 21:712–6. 10.1016/j.phymed.2013.10.026 24268296

[12] Jiang N Fan LX Yang YJ Liu XM Lin HY Gao L. Antidepressant effects of the extract of *Dendrobium nobile* Lindl on chronic unpredictable mild stress-induced depressive mice. Sheng Li Xue Bao. (2017) 69:159–66. 10.13294/j.aps.2017.0006 28435974

[13] Lu C Lv JW Jiang N Wang HX Huang H. Protective effects of Genistein on the cognitive deficits induced by chronic sleep deprivation. Phytother Res. (2020) 34:846–58. 10.1002/ptr.6567 32115816

[14] Feng L Wu HW Song GQ Lu C Li YH Qu LN. Chronical sleep interruption-induced cognitive decline assessed by a metabolomics method. Behav Brain Res. (2016) 302:60–8. 10.1016/j.bbr.2015.12.039 26747207

[15] Li LS Lu YL Nie J Xu YY Zhang W Yang WJ. *Dendrobium nobile* Lindl alkaloid, a novel autophagy inducer, protects against axonal degeneration induced by Aβ25-35 in hippocampus neurons in vitro. CNS Neurosci Ther. (2017) 23:329–40. 10.1111/cns.12678 28261990 PMC6492701

[16] Zhao FK Wu Q Lu YF Yang WJ Yao XD Zhang MS. The extraction and the improvement of determination method for total alkaloids in *Dendrobium nobile* Lindl. Jo Zunyi Med Univer. (2015) 38:5. 10.14169/j.cnki.zunyixuebao.2015.0127

[17] Antunes M Biala G. The novel object recognition memory: neurobiology, test procedure, its modifications. Cogn Process. (2012) 13:93–110. 10.1007/s10339-011-0430-z 22160349 PMC3332351

[18] Denninger JK Smith BM Kirby ED. Novel object recognition and object location behavioral testing in mice on a budget. J Vis Exp. (2018) 141:10.3791/58593. 10.3791/58593 30531711 PMC6800058

[19] Lu C Lv JW Dong LM Jiang N Wang Y Fan B. The protective effect of 20(S)-protopanaxadiol (PPD) against chronic sleep deprivation (CSD) - induced memory impairments in mice. Brain Res Bull. (2018) 137:249–56. 10.1016/j.brainresbull.2017.12.012 29287793

[20] Wang Q Sun LH Jia W Liu XM Dang HX Mai WL. Comparison of ginsenosides Rg1 and Rb1 for their effects on improving scopolamine-induced learning and memory impairment in mice. Phytother Res PTR. (2010) 24:1748–54. 10.1002/ptr.3130 20564503

[21] Wang Q Zhang YL Li YH Chen SG Gao JH Chen YX. The memory enhancement effect of Kai Xin San on cognitive deficit induced by simulated weightlessness in rats. J Ethnopharmacol. (2016) 187:9–16. 10.1016/j.jep.2016.03.070 27103112

[22] Zhou BZ Li MX Cao XY Zhang QL Liu YT Ma Q. Phenylethanoid glycosides of Pedicularis muscicola Maxim ameliorate high altitude-induced memory impairment. Physiol Behav. (2016) 157:39–46. 10.1016/j.physbeh.2016.01.037 26825251

[23] Heckman PRA Roig Kuhn F Meerlo P Havekes R. A brief period of sleep deprivation negatively impacts the acquisition, consolidation, and retrieval of object-location memories. Neurobiol Learn Mem. (2020) 175:107326. 10.1016/j.nlm.2020.107326 33059032

[24] Akbarabadi A Niknamfar S Vousooghi N Sadat SMS Toolee H Zarrindast MR. Effect of rat parental morphine exposure on passive avoidance memory and morphine conditioned place preference in male offspring. Physiol Behav. (2017) 184:143–9. 10.1016/j.physbeh.2017.11.024 29174820

[25] Morris R. Developments of a water-maze procedure for studying spatial learning in the rat. J Neurosci Methods. (1984) 11:47–60. 10.1016/0165-0270(84)90007-4 6471907

[26] Li YF Li F Gong QH Wu Q Shi JS. Inhibitory effects of Dendrobium alkaloids on memory impairment induced by lipopolysaccharide in rats. Planta Med. (2011) 77:117–21. 10.1055/s-0030-1250235 20717874

[27] Xie G Huang X Li H Wang P Huang P. Caffeine-related effects on cognitive performance: roles of apoptosis in rat hippocampus following sleep deprivation. Biochem Biophys Res Commun. (2020) 534:632–8. 10.1016/j.bbrc.2020.11.029 33213844

[28] Zhang K Lian NQ Ding R Guo CL Dong X Li YY. Sleep deprivation aggravates cognitive impairment by the alteration of hippocampal neuronal activity and the density of dendritic spine in isoflurane-exposed mice. Front Behav Neurosci. (2020) 14:589176. 10.3389/fnbeh.2020.589176 33328920 PMC7719754

[29] Paulus MP Dulawa SC Ralph RJ Geyer MA. Behavioral organization is independent of locomotor activity in 129 and C57 mouse strain. Brain Res. (1999) 835:27–36. 10.1016/S0006-8993(99)01137-3 10448193

[30] Fernandes SL Patti CL Zanin KA Fernandes HA Tufik S Andersen ML. Sleep deprivation impairs emotional memory retrieval in mice: influence of sex. Prog Neuropsychopharmacol Biol Psychiatry. (2012) 38:216–22. 10.1016/j.pnpbp.2012.03.014 22521334

[31] Sutcliffe JS Marshall KM Neill JC. Influence of gender on working and spatial memory in the novel object recognition task in the rat. Behav Brain Res. (2006) 177:117–25. 10.1016/j.bbr.2006.10.029 17123641

[32] Pitsikas N Rigamonti AE Cella SG Muller EE. The non-NMDA receptor antagonist NBQX does not affect rats performance in the object recognition task. Pharmacol Res. (2002) 45:43–6. 10.1006/phrs.2001.0898 11820860

[33] Antonio FB Millón C Gago B Laura GD Noelia CC Coveñas R. Galanin (1-15)-fluoxetine interaction in the novel object recognition test. Involvement of 5-HT1A receptors in the prefrontal cortex of the rats. Neuropharmacology. (2019) 155:104–12. 10.1016/j.neuropharm.2019.05.023 31128121

[34] Nikolaos P. The role of nitric oxide in the object recognition memory. Behav Brain Res. (2015) 285:200–7. 10.1016/j.bbr.2014.06.008 24933185

[35] Ahmed A Zeng GR Jiang DJ Lin HY Azhar M Ahsana DF. Time-dependent impairments in learning and memory in Streptozotocin-induced hyperglycemic rats. Metab Brain Dis. (2019) 34:1431–46. 10.1007/s11011-019-00448-7 31286327

[36] Mai WL Wang Q Liu XM Li YH Chen SG Wang LW. Significant Parameters in evaluation of spacetaneous activity in mice. Acta Lab Anim Sci Sinica. (2008) 3:172–5

[37] Kwon KJ Lee EJ Kim MK Jeon SJ Choi YY Shin CY. The potential role of melatonin on sleep deprivation-induced cognitive impairments: implication of FMRP on cognitive function. Neuroscience. (2015) 301:403–14. 10.1016/j.neuroscience.2015.05.079 26047724

[38] Zhang YL Wang Q Chen HL Liu XM Lv K Wang TM. Involvement of cholinergic dysfunction and oxidative damage in the effects of simulated weightlessness on learning and memory in rats. Biomed Res Int. (2018) 2018:2547532. 10.1155/2018/2547532 29581965 PMC5822892

[39] Miyazaki S Uchida S Mukai J Nishihara K. Clonidine effects on all-night human sleep: Opposite action of low- and medium-dose clonidine on human NREM–REM sleep proportion. Psychiatry Clin Neurosci. (2004) 58:138–44. 10.1111/j.1440-1819.2003.01207.x 15009817

[40] Colavito V Fabene PF Grassi ZG Pifferi F Lamberty Y Bentivoglio M. Experimental sleep deprivation as a tool to test memory deficits in rodents. Front Syst Neurosci. (2013) 7:106. 10.3389/fnsys.2013.00106 24379759 PMC3861693

[41] Zhu L Zhang R Li TL. Effects of Acanthopanax on learning and memory and monoamine neurotransmitters in hippocampus of sleep deprived rats. Chin J Traditional Med Formulae. (2012) 18:4. 10.1016/J.BRAINRESBULL.2021.06.013 34153383

[42] Silva RH Abílio VC Takatsu AL Kameda SR Grassl C Chehin AB. Role of hippocampal oxidative stress in memory deficits induced by sleep deprivation in mice. Neuropharmacology. (2004) 46:895–903. 10.1016/j.neuropharm.2003.11.032 15033349

[43] Ennaceur A Delacour J. A new one-trial test for neurobiological studies of memory in rats. 1: behavioral data. Behav Brain Res. (1988) 31:47–59. 10.1016/0166-4328(88)90157-X 3228475

[44] Farajdokht F Vatandoust SM Hosseini L Fekri K Rahigh Aghsan S Majdi A. Sericin protects against acute sleep deprivation-induced memory impairment via enhancement of hippocampal synaptic protein levels and inhibition of oxidative stress and neuroinflammation in mice. Brain Res Bull. (2021) 174:203–11. 10.1016/j.brainresbull.2021.06.013 34153383

[45] Alzoubi KH Khabour OF Rashid BA Damaj IM Salah HA. The neuroprotective effect of vitamin E on chronic sleep deprivation-induced memory impairment: the role of oxidative stress. Behav Brain Res. (2012) 226:205–10. 10.1016/j.bbr.2011.09.017 21944940

[46] Villafuerte G Miguel PA Rodríguez EM Machado S Manjarrez E Arias CO. Sleep deprivation and oxidative stress in animal models: a systematic review. Oxid Med Cell Longev. (2015) 2015:234952. 10.1155/2015/234952 25945148 PMC4402503

